# Spinal Cord Injury and Bladder Dysfunction: New Ideas about an Old Problem

**DOI:** 10.1100/tsw.2011.26

**Published:** 2011-01-18

**Authors:** Célia Duarte Cruz, Francisco Cruz

**Affiliations:** ^1^ Institute of Histology and Embryology, Faculty of Medicine, University of Porto, Porto, Portugal; ^2^ IBMC-Instituto de Biologia Molecular e Celular, University of Porto, Porto, Portugal; ^3^ Department of Urology, Hospital de S. João, Porto, Portugal

**Keywords:** bladder, lower urinary tract, spinal cord injury

## Abstract

Control of the lower urinary tract (LUT) requires complex neuronal circuits that involve elements located at the peripheral nervous system and at different levels of the central nervous system. Spinal cord injury (SCI) interrupts these neuronal circuits and jeopardizes the voluntary control of bladder function. In most cases, SCI results in a period of bladder areflexia, followed by the emergence of neurogenic detrusor overactivity (NDO). Only recently, researchers have started to have a clearer vision ofthe mechanisms of SCI-induced changes affecting LUT control. For example, changes in the urothelium have recently been described and proposed to play a role in NDO. As such, a better understanding of NDO has generated new opportunities to investigate novel therapeutic approaches for NDO.In the present paper, we aim to update recent data concerning SCI-induced LUT dysfunction and therapeutic approaches commonly used to deal with NDO. We make a brief description of LUT control and changes occurring after SCI, and refer to new therapeutic options, including vanniloids and botulinum toxin. Finally, we discuss mechanisms of spinal cord repair, an interesting and very active area of investigation that has obtained some promising results in the recovery of LUT control.

## INTRODUCTION

The normal function of the lower urinary tract (LUT) requires a fine coordination between the activities of the urinary bladder and the urethral sphincter.This coordination depends on the activation of sophisticated neuronal circuits involving peripherally and centrally located neurons[1]. In addition, unlike other visceral organs, LUT function is under the control of cortical centers, which encode the appropriate location and timing for micturition. In fact, LUT function is regulated by behavior learned during maturation of the central nervous system (CNS). The considerable complexity of regulatory neuronal mechanisms renders LUT function sensitive to a variety of injuries and diseases, particularly those affecting the nervous system. Such is the case of spinal cord injury (SCI), which will be the focus of the present review. Other neurological pathologies, including multiple sclerosis and Parkinson's disease, also cause a significant impact on LUT function, butwill not be addressed in the present review. Interested readers may find appropriate information elsewhere[2,3].

SCI may result from traumatic (such as spinal transection or contusion) or ischemic insult occurring at different levels of the spinal cord[4]. These injuries generate a vast array of events that compromise the integrity of the neuronal pathways running in the spinal cord. Some animal models of SCI have been developed over the years, including chemical lesions, induction of ischemia, complete or incomplete transection, and graded contusion or compression of the cord. These experimental models take into consideration the extension and completeness of SCI and mimic several aspects of human pathologies[5,6,7,8,9]. Irrespective of its cause, SCI causes substantial anatomical reorganization of spinal circuits, affects the imbalance between excitatory and inhibitory inputs, and impairs certain cellular functions that may jeopardize the survival of spinal neurons[10]. Therefore, SCI significantly impairs micturition and results, initially, in areflexiaor detrusor underactivityand, later, in neurogenic detrusor overactivity (NDO), depending the level of the lesion. Here, we will address the effects of SCI on LUT function and revisit some of the available therapeutic strategies designed to overcome SCI-induced LUT dysfunction.

## MICTURITION CONTROL IN SPINAL-INTACT ANIMALS

### Peripheral Innervation of LUT

Innervation of the LUT arises from three sets of nerves: (1) pelvic, (2) hypogastric, and (3) pudendal[1,11]. The three nerves convey both motor and sensory input onto the LUT[12]. Whereas the pelvic nerve provides an excitatory input to the bladder, the hypogastric nerve provides inhibitory input to the bladder and excitatory input to the bladder outlet. The pudendal nerve innervates the striated muscle of the sphincter and the pelvic floor[13,14].

### *Sensory Innervation*

Sensory afferent fibers run in the three sets of peripheral nerves and transmit information to the CNS about bladder filling. The majority of bladder sensory fibers run in the pelvic nerve and their cell bodies are located in the sacral dorsal root ganglia (DRG) in humans, whereas they are situated in the L5-S1 DRGin the rat, with a peak in L6. Sensory fibers running in the hypogastric nerve have their cell bodies situated in T12-L2 DRG.

In the bladder, afferent axons may be found throughout the bladder wall and, in experimental animals, are more abundant in the trigone than in the bladder body and dome. Bladder sensory fibers are arranged in a dense suburothelial and muscular plexus, appearsurrounding blood vessels and in the vicinity of intramural parasympathetic ganglia[15,16].Some of the sensory fibers may invade the overlaying urothelium and reach the bladder inner surface[17]. In the urethra, sensory afferents are also present in the suburothelium and muscular layer[18,19].

The vast majority of sensory afferents innervating the LUT, and the bladder in particular, are small myelinated (Aδ fibers) orunmyelinated (C fibers) axons. Bladder afferents, C fibers in particular, may also beidentified by their neuropeptide content and expression of transient receptor potential vanilloid 1 (TRPV1)[17].In addition, bladder afferents are also known to express the tropomyosin-related kinase A (TrkA), the high-affinity receptor for nerve growth factor (NGF), and TrkB, the high-affinity receptor of brain-derived neurotrophicfactor (BDNF)[20,21,22] and purinergic receptors ($P2X_3$, $P2X_2$, P2Y)[4], among many others.

### *Parasympathetic Innervation*

Parasympathetic neurons regulate the contraction of the detrusor. They are located in the sacral parasympathetic nucleus of the sacral spinal cord, and send long dendrites along the lateral funiculus, lateral dorsal horn, and dorsal commissure[23,24]. Their axons run in the pelvic nerve and synapse with postganglionic nerve fibers in the vesical ganglia located outside the bladderin the rat or in the intramural ganglia in humans, guineapig, and mice[25,26]. Parasympathetic ganglia contain, in addition to parasympathetic fibers, sympathetic and sensory nerves that may modulate parasympathetic transmission. The main neurotransmitter involved in parasympathetic signaling in the LUT is acetylcholine.In addition, parasympathetic nerve fibers may innervate the urethra and release nitric oxide, thereby inducing the relaxation of the outflow region[25,26].

### *Sympathetic Innervation*

Most sympathetic neurons are located in the thoracolumbar spinal cord. Their axons run in the hypogastric and pelvic nerves. Sympathetic innervation of the LUT is responsible for counteracting the effects of parasympathetic signaling. Thus, the release of noradrenaline leads to relaxation of the detrusor and contraction of the bladder outlet[25,26].

### *Somatic Innervation*

Somatic motoneurons are located in the Onuf's nucleus, an area located in the ventral horn of the sacral spinal cord. These neurons send their axons through the ventral roots into the pudendal nerve and innervate the external urethral sphincter. During urine accumulation, activation of the pudendal nerve maintains the contraction of the sphincter[25,26].

## Spinal Pathways Involved in LUT Control

Afferent neurons innervating the LUT send their central projections to specific areas of the spinal cord. The location of central projections and their relation to spinal interneurons has been extensively studied using axonal tracing methods and analyzing the expression of the immediate early gene *c-fos*[27,28,29,30,31]. Spinal neurons involved in the regulation of LUT function are located in the dorsal commissure, superficial dorsal horn, and parasympathetic nucleus[27,28,32]. While some interneurons send projections to the brain, others participate in local circuits regulating spinal segmental reflexes[33]. Pharmacological studies have demonstrated that glutamate is the excitatory transmitter regulating local neuronal circuitry, while inhibitory interneurons release γ-aminobutyric acid (GABA) and glycine[34,35]. Interestingly, in the dorsal horn and in the dorsal commissure, an overlap of the central projections of bladder and urethral afferents has been observed[36]. This suggests that those spinal cord areas may act as sites of viscerosomatic integration necessary for proper coordination of bladder and sphincter function. More detailed information regarding the peripheral innervation and spinal pathways involved in LUT function can be found elsewhere[1,4,25,26].

## The Urothelium as a Participant of Micturition Regulation

The urothelium, the epithelial lining of the urinary bladder, has been traditionally viewed as an impermeable barrier that prevents the contact of urine with underlying tissues. Nowadays, there is increasing evidence that urothelial cells may also participate in bladder sensory mechanisms, serving as primary transducers of physical and chemical stimuli[37,38,39]. The evidence for this is compelling. Indeed, it is known that urothelial cells express a variety of sensory receptors, including TRPV1[40], muscarinic and nicotinic receptors[41,42,43], and bradykinin receptors[44], which enables them to respond to several stimuli. In addition, upon stimulation, urothelial cells release chemical mediators like ATP[40,45], acetylcholine[46], and nitric oxide[47]. As urothelial cells are in close proximity with afferent and efferent nerve fibers[48,49,50], it is likely that they influence the activity of those nerve fibers coursing in the bladder wall. Therefore, urothelial cells may modulate LUT sensation and activity.

## SupraspinalPathways Regulating Micturition

The identification of supraspinal structures involved in micturition control was pioneered by Frederick James Fitzmaurice Barrington, who identified a structure in cats located in the dorsolateral pontinetegmentum crucial for micturition control[51,52].Barrington observed that bilateral lesions of this area led to urinary retention. This region is now known as the pontinemicturition center (PMC), Barrington's nucleus, or M-region. Further studies using anterograde and retrograde neurotracers and ultrastrucutral analysis established the connections between the PMC and sacral parasympathetic motoneurons located in the dorsal commissure and sacral intermediolateral cell column[53,54,55,56].

The identification of supraspinal structures involved in micturition control was alsoachieved by injection of pseudorabies virus (PRV) in experimental animals, by using brain-lesioningapproaches and by imaging techniques in human patients. PRV is a neurotropic virus that is transported across multiple synapses. Thus, following injection into the urinary bladder or urethra, PRV was detected at the same spinal cord locations (superficial laminae, dorsal commissure, and parasympathetic nucleus), suggesting an overlap of spinal pathways involved in LUT function[32,36,57,58,59,60]. In addition, PRV was also detected in a series of supraspinal structures, including the PMC, the medulary raphe nucleus, locus coeruleus, periaqueductal gray (PAG), and the A5 nucleus[32,57,58,60]. Specific areas of the hypothalamus (such as the para- and periventricular nuclei and the medial preoptic area) and the medial frontal cortex were also shown to be infected by bladder-delivered PRV[32,57,58,60]. As in the spinal cord, injection of the PRV into the bladder or urethra results in a similar pattern of PRV labeling, further supporting the existence of common neuronal pathways regulating bladder and urethra function[36,58,59,61]. Data obtained in brain-lesioning experiments have corroborated the PRV studies. For example, bilateral impairment of the locus coeruleus leads to micturition blockade, whereas electrical or chemical stimulation of that supraspinal structure causes bladder contraction and urethra relaxation, resulting in bladder emptying[60,62,63].

More recently, studies utilizing neuroimaging techniques in humans provided evidence that came to agree with data obtained in rats. Nevertheless, published work focusing on brain control of LUT function is still relatively scarce, but it is likely that, with the enhancement of functional brain-imaging techniques, new data will be made available in the near future. The first study using functional brain imaging in human volunteers was published by Fukuyama et al.[64], who observed neuronal activation in the upper pons, left sensorimotor cortex, right frontal cortex, and bilateral supplementary areas during micturition. This team of researchers used single photon computerized tomography (SPECT), an imaging technique using γ-rays that lacks proper spatial resolution. In 1997, Blok and coworkers used positron emission tomography (PET) to study brain activity in male volunteers during bladder filling and voiding[65]. This imaging technique relies on the emission of positrons by decaying isotopes and, like SPECT, requires the administration of a radioactive substance to patients. In comparison with SPECT, PET has a better spatial and temporal resolution.Results obtained showed increased activity in the right dorsomedialpontinetegmentum, the PAG, the hypothalamus, and the right inferior frontal gyrus during micturition[65]. The importance of some of these areas, like the PAG, was previously established in cats in which it was shown that sacral afferents send their projections to this supraspinalstructure[66]. The demonstration of increased activity of the PAG in humans proved the similarity between the neuronal control of LUT function in humans and cats.

Information obtained with PET studies may be complemented using functional magnetic resonance imaging (fMRI). This new imaging technique has the advantage of being less invasive than SPECT and PET as it circumvents the use of radioactivity,although the signal/noise ratio is still low.By combining data obtained with PET and fMRI, it was possible to produce maps illustrating the brain regions activated during LUT function[1,67,68,69] and the concept of the "bladder control matrix" was born. This diagram establishes the network and the direction of connectivity of brain structures[65,70] actively involved in micturition control[69,71].The brain control matrix proposes that during storage, ascending afferent input reaches the PAG and is relayed via the hypothalamus and thalamus to the anterior cingulate cortex, to the insula, and to the prefrontal cortex. The prefrontal cortex,hypothalamus, insula, and anterior cingulated cortex inhibit the PAG[1,67,68,69]. As the PAG is connected to the PMC, this structure is also inhibited until a decision to void is made. When this happens, PAG inhibition by the prefrontal cortex is interrupted. The hypothalamus also stimulates the PAG that, in turn, excites the PMC. The PMC sends excitatory motor input to the sacral spinal cord, resulting in detrusor contraction and relaxation of the urethra[69]. Interestingly, PET and fMRI studies have also demonstrated the activation of the cerebellum and basal ganglia during LUT function, although the relevance of this remains unclear.

## SPINAL CORD INJURY

### Molecular Mechanisms of Spinal Injury

The most common animal model of SCI involves mechanical trauma of the cord, usually resulting from a single mechanical insult. SCI-induced cord changes occur in a two-step process. The first step refers to the direct tissue damage and includes acute compression, laceration, or shearing of the cord, which results in profound histological changes[72,73]. The second step refers to the destructive events initiated by the physical trauma. These secondary events comprise vascular changes (edema, ischemia, and hypoxia), biochemical responses (production of free radicals, lipid peroxidation, and decreased ATP production), and cellular responses (invasion by immune cells, activation of glial cells, and apoptosis of neurons and glial cells)[72,73].

The primary effect of SCI is the interruption of ascending and descending connections between the spinal cord and supraspinal centers. Spinal segments caudal to SCI are left with little or no contact with the brain and lesioned neuronal processes undergo Wallerian degeneration[74]. In addition, at the injury site, glial cells, activated by cellular debris and cytokines, initiate the synthesis of a scar that closes the wound[75]. This glial scar prevents the growth and rewiring of surviving processes. Some of the main components of the glial scar are chondroitin sulfate proteoglycans (CPGs), synthesized by activated astrocytes. CPGs are a large family of glycoproteins and include neurocan, versican, aggrecan, brevican, phosphacan, and NG2[74]. CPGs are known to inhibit neurite outgrowth both in *in vitro* and *in vivo*[76,77]. Spontaneous regrowth of surviving processes is also hampered by specific proteins originated by lesion of myelinated axons running in the cord. This group of myelin-derived inhibitory proteins includes three members: NOGO, myelin-associated glycoprotein (MAG), and oligodendrocyte myelin glycoprotein (OMgp)[78,79,80]. More recently, other molecules have been shown to be up-regulated after SCI at the site of lesion and seem to contribute to the limited capacity of self-regeneration of the CNS, although more studies are clearly needed. Examples of these proteins include Semaphorin III, Ephrin proteins, and Slit proteins[81,82,83,84].

### Changes in the Neuronal Pathways Innervating the LUT

The consequences of SCI on LUT function depend on the level, duration, and severity of the lesion. Thoracic and cervical injuries are the most common injuries among SCI patients[72]. In this case, SCI is followed by a period of bladder areflexiaand urinary retention, the duration of which varies according to the species, from weeks to months. Eventually, a new micturition reflex emerges,exclusively located at the spinal cord and not dependent on supraspinal structures. Moreover, while in normal conditions, the micturition reflex is initiated by activation of low-threshold Aδ fibers, this does not happenin SCI animals. Instead, the spinal micturition reflex is totally dependent on high-threshold capsaicin-sensitive Cfibers. Accordingly, it has been demonstrated that systemic administration of capsaicin reduces bladder overactivity in SCI rats[85,86].

Despite this new micturition reflex, voiding is typically inefficient due to detrusor-sphincter-dyssinergia (DSD), a condition characterized by simultaneous contraction of the detrusor and urethral sphincter. Ineffective bladder emptying causes the accumulation of large residual volumes of urine and bladder hypertrophy[87]. The large amounts of urine accumulated in the bladder results in high intravesical pressure, deleterious to the upper urinary tract and a life-threatening situation[88]. In addition, the high intravesical pressures may also trigger episodes of autonomic dysreflexia, a common clinical syndrome observed in SCI patients[89]. Autonomic dysreflexiais typically seen in cases of high cervical spinal cord lesions. Episodes are triggered by noxious stimulation of body areas and viscera, like the urinary bladder, innervated by spinal segments below the injury level.Sensory inputs arriving to the spinal cord trigger a discharge of sympathetic neurons located in the thoracolumbar spinal cord. Because there is lack of supraspinal inhibition, an enhanced sympathetic output results in exaggerated vasoconstriction in the lower half of the body, which leads to a rapid blood mobilization from the mesenteric bed into the systemic circulation and a dramatic rise in blood pressure. In turn, baroreceptors located in the carotid arteries are activated and induce vasodilation and profuse sweating in the upper body,along with bradycardia[90].Rapid decrease of blood pressure with calcium channel blockers, such asnifedipine,is recommended along with the removal of the trigger stimuli. Immediate emptying of the bladder may be, therefore, necessary[91].

The emergence of the new micturition reflex, located below the lesion site, results from a combination of factors, including (1) loss of bulbospinal inhibitory input, (2) strengthening of existing spinal synapses or formation of new ones due to neuronal sprouting, (3) alterations in neurotransmitter metabolism and release, (4) changes in afferent input generated in the bladder, and/or (5) alterations in the synthesis and release of neurotrophic factors at the peripheral and central nervous system[92]. The key event seems to be the increased expression of neurotrophic factors in the neuronal micturition pathways, particularly NGF. In fact, in SCI animals, the levels of NGF are strongly up-regulated[93,94] and intrathecal neutralization of this neurotrophin improved bladder functions[6,7].

Appearance of the spinal micturition reflex is accompanied by noteworthy changes in the morphology, neurofilament and neuropeptide content, and electrophysiological properties of bladder afferents. Chronic SCI in rats resulted in hypertrophy of bladder afferent neurons as reflected by an increase in cell diameter[95] and the number of bladder afferents expressing neurofilaments[96]. An up-regulation of the calcitonin gene-related peptide (CGRP)has alsobeen found at the spinal cord level both in rats[97] and in humans[98], suggesting that CGRP is synthesized in the cell bodies and actively transported to the central terminals of sensory afferents. As for other neuropeptides, a strong increase in the expression of pituitary adenylatecyclase–activating polypeptide (PACAP),both at central terminals and in the cell body of bladder afferents,has been reported[99].As PACAP has been shown to facilitate bladder reflex contractions in spinal-intact rats[100,101,102], it is likely that in SCI animals, PACAP may exacerbate the excitability of bladder afferents and contribute to bladder dysfunction. Another neuropeptide that has attracted attention is galanin, which has been proposed to have an inhibitory effect on the cholinergic transmission in human bladder strips[103]. Like PACAP, the expression of galanin in the cell body of bladder afferents and at their central terminals is up-regulated[94].

SCI also results in alterations in the electrophysiological properties of bladder afferents. Indeed, whereas the majority of bladder sensory neurons collected from spinal-intact animals display high-threshold tetrodotoxin (TTX)-resistant currents, most bladder afferents from SCI rats exhibit low-threshold TTX-sensitive action potentials[104]. Because increased TTX-sensitive sodium currents are increased in parallel with suppression of A-type potassium currents following SCI[87,104,105], bladder sensory afferents become hyperexcitable.

SCI may also induce changes at the spinal cord level, reflecting the central reorganization of LUT neuronal pathways due to the formation of new synapses and/or to alteration of pre-existing ones[106]. This may result from sprouting known to occur in the peptidergic[97,106,107]and nonpeptidergicpopulation of bladder afferents[108]in chronic SCI rats. This is supported by increased expression of growth-associated protein (GAP) 43[108,109,110]. The high spinal levels of PACAP and galaninfound in SCI animals further demonstrate the occurrence of sprouting of spinal afferents[94,99]. Moreover, following SCI, a marked difference can be seen when analyzing the spinal distribution of sensory afferents expressing vasoactive intestinal polypeptide (VIP). Indeed, in spinal-intact rats, VIP-IR (immunoreactivity) afferents display a periodic distribution along the spinal segments receiving bladder-generated sensory input. After SCI, VIP-IR afferents extend and rearrange themselves, producing a continuous band of VIP-positive axons[102,106].

Apart from structural changes occurring at the spinal neuronal LUT pathways, neurochemical alterations may also occur. Accordingly, the mRNA levels of glutamic acid decarboxylase *(*GAD) 67, the enzyme that catalyzes the conversion of glutamate to GABA, are strongly decreased in the spinal cord of SCIrats[111,112]. This could mean that spinal inhibitory mechanisms are compromised in SCI rats, contributing to bladder dysfunction. Moreover, changes in glutamatergic transmission have also been reported[113,114], implying that glutamate-dependent mechanisms are also modified in consequence of SCI, and there is an imbalance between excitatory and inhibitory transmission in the spinal LUT pathways**.**Also important, it has been demonstrated that spinal activation of the extracellular signal-regulated kinase 1 and 2 (ERK 1/2) pathway is strongly up-regulated in SCI rats[5,115]. ERK blockade improved bladder function in these animals, suggesting that this signaling cascade participates in NDO[5,115]. As spinal ERK activation is modulated by glutamate and GABA[116,117,118], the importance of ERK in SCI rats further supports the existence of an imbalance of glutamatergic and GABAergic transmission.

## Changes in the Bladder

Following SCI, a number of alterations occurring in the urothelium have been described, which may interfere with the permeability of the urothelial barrier as well as its sensory function. One of the major changes observed is the occurrence of disruption of the urothelium accompanied by reduction in the transepithelial resistance. This happened as early as 2 h after SCI,but some recovery of transepithelial resistance occurred at later time points[119].Changes in the morphology of the urothelium have also been detected, including disappearance of apical cells in some areas, disorganization of cell layers, and reduction of cellular volume[119]. Treatment of animals, prior to SCI, with capsaicin and hexamethonium, an antagonist of the acetylcholine receptor expressed by nerve fibers, affected the transepithelial resistance, further reinforcingthe intimate relation between the urothelium and the nervous system[39,120,121]. Along with morphological changes, the expression of sensory receptors by urothelial cells is also affected by SCI. Recent studies have shown that the expression of the $P2X_2$ receptor in the human urothelium from SCI patients is increased[122], eventually enhancing responses to ATP. Likewise, the expression of TRPV1 in urothelial cells obtained from patients with neurogenic bladder is up-regulated[123]. To our knowledge, the expression of other receptors has not yet been investigated, not even the expression of the high-affinity receptor of NGF, TrkA. This is highly relevant as high levels of NGF have been found in the urine of NDO patients[124,125]. It is possible that TrkA expression may accompany the high NGF levels and beup-regulated, as in rats with cystitis[126], and contribute to urothelial dysfunction.

Urothelial cells from SCI rats also present increasedheightened release of excitatory mediators, including prostaglandins[127], ATP, and nitric oxide[128], which may contribute to increased excitability of bladder sensory afferents present in the suburothelial region or among urothelial cells. Another major consequence of SCI is increased expression of connexin 26in the urothelium, an event that is possibly involved in detrusor overactivity, as blockade of gap junctions reduced bladder contractions[129].

One population of bladder cells that has attracted some attention over the years is the interstitial cell (IC) population. This population of cells may be divided into ICs of the detrusor, present in the vicinity of smooth muscle cells, and myofibroblasts (MFs). MFs are long, spindle-like cells present in the suburothelium region[130]. They are connected via connexin 43 and function as a syncitium. MFs are located close to afferent fibers and are sensitive to a variety of stimuli, including variations in local pH and ATP[129,130].Recent studies suggest that they could play a role in the overactive bladder syndrome[129]. Accordingly, it was also demonstrated that the expression of connexin 43 was also up-regulated in the suburothelial region in rats with SCI[129]. Consistent with this, in biopsies from patients with NDO, the expression of the same connexin was increased in the suburothelium, suggesting that, like in the respective animal model, increased gapjunctions linking MFs may play a role in neurogenic bladder dysfunction[131].

## COMMONLY USED THERAPIES FORSCI-INDUCED BLADDER DYSFUNCTION

One of the major concerns reported by SCI patients is loss of bowel and bladder control. In addition, due to DSD, intravesical pressure may reach very high values in these patients, above 40 cm $H_2O$, posing serious threats to the upper urinary tract[132]. Therefore, over the years, several therapeutic strategies have been developed that aim to safeguard the kidneys and maintain quality of life. These treatment options can be divided into two main groups: therapies to promote storage of urine and therapies to facilitate bladder emptying. Both groups include pharmacological treatments and surgical approaches. The most commonly used method consists of reducing the activity of the detrusor and promoting urine removal by clean intermittent catheterization (CIC). This procedure has become a standard management method for SCI patients[132] and comprises several advantages, including improved quality of life[133].

### Anticholinergic Drugs

Bladder contractions are induced by activation of postjunctional muscarinic receptors by acetylcholine released from parasympathetic nerves coursing in the bladder wall. In the human bladder, the main subtypes of muscarinic receptors are the M2 and M3, although the M1 receptor is also located in the prejunctional neuronal terminal[134,135]. Activation of prejunctional M1 receptors in SCI rats has been shown to facilitate the release of acetylcholine, suggesting that NDO may result from enhanced release of acetylcholine[135]. As such, blocking cholinergic transmission with a muscarinic receptor antagonist is still the first option for NDO[134,136,137], as they reduce detrusor reflex activity.The main problem with this therapeutic approach resides with the high doses required, above those recommended by the manufacturers[138,139]. Earlier formulations induced significant sideeffects, but the tolerability of antimuscarinics has been improved by the development of more selective drugs for the bladder and extended-release oral formulations[134].Nevertheless, there is a generalized lack of randomized, placebo-controlled studies on neurogenic bladder treatment. As placebos fail to decrease the deleterious high intravesical pressures registered in SCI patients[140,141,142], most researchers consider it safe to use them only in healthy volunteers[143,144].

### Opioids

Precursors of endogenous opioids and their specific receptors are distributed in many areas of the CNS concerning the regulation of micturition, including the PAG, PMC, and the parasympathetic nucleus in the spinal cord[145]. Agonist binding or blocking of the classical opioid receptors ($\mu$, $\delta$, $\kappa$) interferes with voiding in intact animals and experimental models of bladder dysfunction. Morphine increases bladder capacity in experimental animals and humans[146,147], whereas naloxone stimulates voiding[148].

Administration of δ-receptor agonists, either intrathecally or via intracerebroventricular injection,inhibits voiding contractions[149]. Interestingly, the opioid nociceptin/orphanin, which binds to the NOP receptor[150], inhibits the micturition reflex in patients with neurogenic incontinence, but not in healthy individuals[151].

Opioids have been used scarcely to treat NDO. Intrathecal morphine may reduce detrusor overactivity, but patients complain about nausea, pruritus, among other side effects[152]. Tramadol, widely used as an analgesic, is, by itself, a very weak μ-receptor agonist, but can be metabolized in several compounds, some of which may be as effective as morphine. Studies show that, in rats, tramadol can abolish detrusor overactivity caused by activation of the dopamine receptor[153] or by cerebral infarction[154]. Moreover, it was recently shown that following intravenous administration of U-50488, a κ-receptor agonist, DSD was reduced and voiding efficiency improved in SCI rats[155]. Finally, in a randomized, placebo-controlled, double-blind study, SCI patients treated with nociceptin/orphanin presented improvement of bladder function[156]. Further studies are warranted to explore the use of opioids to treat micturition impairment in SCI patients.

## Vanniloids

In cases in which bladder dysfunction is refractory to oral medication, other options may be chosen. Intravesical administration of vanilloid solutions, such as capsaicin or its ultrapotent analogue resiniferatoxin (RTX), has been thoroughly investigated in the past. The underlying principle of using intravesicalvanilloids resides in the demonstration that capsaicin-sensitive Cfibers, expressing the TRPV1 receptor, were important for SCI-induced bladder dysfunction[86,96,106,157].In addition, increased TRPV1 expression has been detected in urothelial cells and nerve fibers in the bladder of SCI patients[158]. In experimental animals with chronic SCI, intravesical administration of capsaicin or RTX resulted in improvement of bladder function[159]. In human SCI patients, both neurotoxins have also been used with good results[160,161,162,163]. However, the high intravesical pressures recorded in SCI patients were not changed after intravesicalvanilloids[164], although there were improvements on detrusor overactivity and urinary incontinence[88,160,164,165]. Another alternative to deliver RTX is via an intrathecal injection. The amounts of RTX injected into the intrathecal space were very small and it was shown that intrathecal injections of RTX led to a reduction of detrusor overactivity in a dose-dependent manner[5]. As in other studies[166,167,168], sideeffects were negligible. As such, intrathecal administration of RTX may be a useful approach to complement or, in some cases, replace current pharmacological therapies.

Despite the number of studies reporting the use of capsaicin and RTX to induce TRPV1 desensitization and consequent inactivation of TRPV1-expressing fibers, the fine mechanisms of TRPV1 desensitization are still mostly unknown. The current working model postulates that binding of vanilloids to the receptor in its phosphorylated state induces a massive entry of $Ca^{2+}$ into the cell[169,170]. The increased intracellular concentration of $Ca^{2+}$ leads to activation of calcineurin, a phosphatase thatreleases TRPV1 from its phosphorylated state[170,171]. As such, the allosteric connection between TRPV1 and phosphatidylinositol-4,5-bisphosphate(PIP2) is disrupted and PIP2 is cleaved by phospholipase C into diacylglycerol and inositol 1,4,5-triphosphate[172]. This results in ATP displacement of the N-terminal portion of TRPV1, allowing binding of complex $Ca^{2+}$-calmodulin to both the N- and C-terminus of TRPV1, ultimately desensitizing the receptor[172]. Nevertheless, the use of vanilloids in clinical practice is not a feasible option mostly due to the pungency[160,161] and lack of stability of vanilloid solutions[158]. There is an obvious need for specific and stable molecules that block TRPV1 activity. In fact, recently, a new oral TRPV1 antagonist, GRC 6211, was shown to reduce bladder overactivity in an animal model of chronic cystitis[173]. The same antagonist has also been tested in rats with SCI, and proved to reduce the high intravesical pressures observed in those animals as well as the frequency of bladder reflex contractions[174].

## BotulinumToxin

Another treatment that is now well established for the treatment of SCI-induced bladder dysfunction is botulinum toxin type A (BTX-A) injections into the bladder wall[175,176,177,178,179], recently introduced as a new treatment for intractable NDO and incontinence[179]. The beneficial effects of BTX-A injections on NDO, incontinence, and quality of life of SCI patients seem to be longlasting[177,180,181,182,183] and there is a strong interest in the development of less-invasive routes for toxin delivery.

BTX-A exerts its effects upon binding to the SV2 protein, a synaptic protein exposed upon neurotransmitter exocytosis. When bound to SV2, BTX-A is internalized. Once within the nerve terminal, the toxin originates a protease that cleaves the synaptosomal-associated protein 25 (SNAP-25), thwarting the assembly of synaptic complexes necessary for synaptic vesicle fusion and neurotransmitter exocytosis[184]. It was initially thought that BTX-A could act directly on urothelial cells[185,186] or on MFs, but none express the SV2 protein and the expression ofconnexin 43 by MFs, which is intense in the suburothelium of SCI patients, was not affected by BTX-A[131]. In fact, recent studies prove that the expression of SV2 and SNAP-25 is restricted to nerve fibers present in the suburothelial region and in the muscular layer[50], excluding a direct action of BTX-A on the urothelium and MFs. SV2 expression was found only in parasympathetic, sympathetic, and primary afferent fibers, as it colocalized with vesicular acetylcholine transporter (VAChT), tyrosine hydroxylase, and CGRP, respectively, indicating that detrusor injections of BTX-A may potentially target all classes of nerve fibers. However, because SV2 expression was more abundant in VAChT-positive parasympathetic fibers, these fibers are likely to be the main target of BTX-A in the human bladder.Accordingly, BTX-A treatment led to a reduction in acetylcholine release[187,188].

In the study by Coelho etal., it was shown that only half of the CGRP-positive sensory fibers expressed SV2[50]. This suggests that the contribution of sensory fibers to the net effect of BTX-A administration is less important than their parasympathetic counterparts. Nonetheless, an effect of BTX-A on sensory fibers cannot be ruled out, as a reduction in noxious bladder input has been reported following intravesical administration of BTX-A[189,190]. Thus, at least in a model of bladder inflammation, BTX-A reduced the release of neuropeptides[191]. In addition, treatment with BTX-A decreased the expression of TRPV1 and the purinergic receptor $P2X_3$ in biopsies from SCI patients, without affecting the overall density of nerve fibers coursing the bladder wall[123]. In intact animals, BTX-A reduced detrusor overactivity induced by intravesical administration of ATP or capsaicin in intact animals[192]. These data suggest that activation of sensory afferents may be impaired by BTX-A. Altogether, the beneficial effects of BTX-A treatment result from impairment of both the afferent and efferent innervations of the bladder, thereby reducing bladder dysfunction[193].

Another reported effect of BTX-A injections in the bladder wall of SCI patients is the reduction of the NGF concentration in the urine[124] and bladder wall of SCI patients[194]. The reasons for this are still unclear and predictably complex. It is possible that the decreased release of acetylcholine by presynaptic terminals following BTX-A injection in the bladder wall[187],which prevents detrusor contractility, impairs NGF synthesis and release. As bladder smooth muscle cells seem to be able to produce and secrete NGF[195,196], BTX-A may affect NGF release into urine and bladder tissues. Since exogenous NGF administration in the bladder may cause detrusoroveractivity[197,198], BTX-A may prevent the excitatory effects of NGF on bladder function.

## Future Trends

Ongoing research is actively looking for alternative methods to treat SCI-induced bladder dysfunction. As mentioned above, GABAergic transmission is impaired in SCI rats[111,112], but treatment with GABA-receptor agonists, like baclofen, reduces detrusor overactivity in SCI animals[111,199]. However, baclofen has a limited therapeutic window, which hinders its use. Therefore, because re-establishment of

GABAergic transmission seems to be of interest for treatment of SCI-induced bladder dysfunction, other ways of increasing GABA-mediated spinal inhibition are being investigated. Recently, it was reported that gene delivery of GAD led to a reduction in detrusor overactivity in SCI rats[200]. The vector injected into the bladder wall was the herpes simplex virus (HSV), which is taken up by sensory afferents. Data obtained in that study indicate that the levels of GAD were restored and accompanied by a reduction in the number of nonvoidingcontractions[200]. This study forwards the possible use of replication-defective HSV vectors transporting genes of interest to treat detrusor overactivity caused by SCI, although this method is still very far from being clinically used in SCI patients.

Another alternative for designing new therapeutic approaches for SCI-induced bladder dysfunction is to target NGF and downstream signaling pathways. This neurotrophic factor has been shown to be involved in this pathology as it has been known to induce alterations in the excitability of bladder afferents[201]. In SCI rats, NGF levels are strongly increased in the bladder[93], but decreased at the spinal cord[94], supporting the involvement of this neurotrophin in NDO. In fact, one should recall that sequestration of NGF has led to improved bladder reflex activity in SCI rats[6,7]. Likewise, exogenous administration of NGF induces changes in bladder function[197,202,203] that overlap to some degree with recordings obtained from SCI rats. In what concerns NGF-mediated downstream signaling cascades, it was also found that blocking the ERK 1/2 pathway improved bladder function in SCI rats[115]. Nevertheless, studies regarding manipulation of NGF levels and NGF-mediated molecular events are scarce, and investigation is clearly needed to explore a possible therapeutic use of NGF sequestration.

## SPINAL CORD REGENERATION

Despite all the available treatments for SCI-induced NDO and incontinence, bladder dysfunction may never be totally overcome as damage to the cord is, in most cases, permanent. One should recall that, unlike the peripheral nervous system, the CNS is not able to regenerate. Therefore, and because SCI is also accompanied by loss of voluntary movement and tactile sensibility, spinal cord repair is an extremely active area of research. Investigation focuses, on one hand, on describing and understanding the consequences of SCI and the reasons for failure of spontaneous repair and, on the other hand, on devising treatments that promote regeneration of lesioned neuronal pathways and functional improvement.

Investigation on spinal cord regeneration after SCI has been focusedon inducing functional restoration of sensorimotor abilities. For that, it is necessary to induce axonal regrowth, to direct growing processes so they can connect with appropriate targets, and to restore original circuitry as best as possible[74]. By targeting the above-mentioned myelin-derived inhibitory proteins, spinal cord repair and improvement of sensorimotor function have been accomplished to some extent. As such, neutralizing the NOGOprotein[204,205,206,207]or blocking the NOGO receptor[208,209] resulted in some degree of spinal cord regeneration and functional improvement. In what concerns MAG and OMgp, the effect of neutralizing these proteins is still to be thoroughly investigated. As for CPGs, it has been shown that interference with the glycosylmoiety of these glycoproteins using chondroitinasemay lead to improved regrowth of spared neuronal processes as well as stimulation of functional recovery[210,211,212,213]. Other SCI repair strategies include the transplantation of undifferentiated or genetically modified cells and infusion with neurotrophicfactors[214,215,216].

The recovery of bladder control has seldom been addressed in the context of SCI repair. Nevertheless, recent studies have started to investigate this matter. Most published papers describe the use of cellular implants or the manipulation of the extracellular environment of the lesion. Indeed, it has been shown that injection of neural stem cells at the lesion site in the cord of SCI rats improved voiding efficiency[217]. Furthermore, the transplantation of neuronal and glial precursors into the lesion site 9 days after SCI resulted in decreased intravesical pressure and reduced detrusor overactivity[218]. Similar results were found when using genetically modified fibroblasts that expressed BDNF and neurotrophin 3 (NT-3)[219], or after transplantation of BDNF- and NT-3–expressing Schwann cells[220].

Olfactory ensheathing cells (OECs) have also been used in some studies. OECs are glial cells derived from the olfactory placode[221]. Because OECs interact with astrocytes and present axonal growth-promoting properties, transplantation of these cells has also been studied as a means to stimulate repair of certain CNSlesions[222,223,224,225,226]. In one study, OECs were injected into the spinal cord in the vicinity of the sacral parasympathetic nucleus in rats submitted to rhizotomy. The authors found that OECs induced afferent re-entry into the dorsal horn and recovery of bladder function[227]. Although rhizotomy cannot be considered a model of SCI, results suggest that the therapeutic potential of OECs to induce spinal repair and improvement of bladder dysfunction should be further investigated.

Manipulating the extracellular environment of the injury site and the composition of the glial scar has also received attention as a potential approach to improve bladder function. CPGs present in the glial scar are produced by reactive astrocytes, which respond to epidermal growth factor (EGF). Chronic blockade of the EGF receptor, achieved with osmotic pumps,resulted in inactivation of astrocytes and improvement of sensorimotor and bladder functions[227]. Chondroitinase has also been tested alone[228] or in combination with cellular transplantation of OECs and Schwann cells[229] and, in both cases, led to recovery of bladder function in rats with SCI. Although it is tempting to infer that spinal cord repair strategies will be successfully used to treat SCI-induced bladder dysfunction, more studies are clearly needed. Approaches tested to promote recovery of sensorimotor function should be furtherinvestigated to assess their efficacy in regaining LUT control.

## CONCLUSION

It is well established that SCI causes a significant impact in bladder function. Our knowledge about the molecular mechanisms ruling SCI-induced bladder dysfunction has improved over the years. This allowed the development of more effective therapies, albeit still with important sideeffects. However, much remains to be investigated, and there is a strong need for further improved understanding of the neurochemical, organizational, and electric changes occurring in the LUT following SCI. Available therapies still produce important secondary effects and investigators are actively working on designing new approaches. The use of vanilloids initially yielded some promising results, but vanilloids were soon disregarded due to the pungency and poor stability of the solutions. In contrast, several papers report long-lasting improved bladder function after botulinum toxin injectionswith little side effects. It is likely that BTX-A will be approved in a near future for NDO treatment. Other strategies, including gene therapy, are also under active investigation and may become important therapeutic options to use in future studies. Moreover, future studies will also focus on NGF and NGF-mediated events, as this neurotrophinseems to be a key factor modulating neuronal pathways regulating LUT function. Lastly, another area of intense investigation is SCI repair. There is more information regarding the complex cellular and molecular events of SCI and this has led to the development of new approaches to induce spinal cord regeneration, sensorimotor recovery, and improvement of bladder function. It is likely that, in the future, the management of NDO will have more numerous and effective options.

## ACKNOWLEDGMENTS

Financial support came from InComb FP7 Health Project #223234.
